# miR-29b-3p Inhibitor Alleviates Hypomethylation-Related Aberrations Through a Feedback Loop Between miR-29b-3p and DNA Methylation in Cardiomyocytes

**DOI:** 10.3389/fcell.2022.788799

**Published:** 2022-04-11

**Authors:** Fang Wu, Qian Yang, Yaping Mi, Feng Wang, Ke Cai, Yawen Zhang, Youhua Wang, Xu Wang, Yonghao Gui, Qiang Li

**Affiliations:** ^1^ Translational Medical Center for Development and Disease, Shanghai Key Laboratory of Birth Defect Prevention and Control, NHC Key Laboratory of Neonatal Diseases, Institute of Pediatrics, Children’s Hospital of Fudan University, National Children’s Medical Center, Shanghai, China; ^2^ Cardiovascular Center, NHC Key Laboratory of Neonatal Diseases, Children’s Hospital of Fudan University, National Children’s Medical Center, Shanghai, China; ^3^ Department of Neonatology, Shanghai General Hospital, Shanghai Jiao Tong University School of Medicine, Shanghai, China; ^4^ Department of Cardiology, Longhua Hospital, Shanghai University of Traditional Chinese Medicine, Shanghai, China; ^5^ Cancer Institute, Fudan University Shanghai Cancer Center, Shanghai, China

**Keywords:** DNA methylation, miR-29b-3p, DNA methyltransferases, congenital heart disease, zebrafish, proliferation

## Abstract

As a member of the miR-29 family, miR-29b regulates global DNA methylation through target DNA methyltransferases (DNMTs) and acts as both a target and a key effector in DNA methylation. In this study, we found that miR-29b-3p expression was inversely correlated with DNMT expression in the heart tissues of patients with congenital heart disease (CHD), but whether it interacts with DNMTs in cardiomyocytes remains unknown. Further results revealed a feedback loop between miR-29b-3p and DNMTs in cardiomyocytes. Moreover, miR-29b-3p inhibitor relieved the deformity of hypomethylated zebrafish and restored the DNA methylation patterns in cardiomyocytes, resulting in increased proliferation and renormalization of gene expression. These results suggest mutual regulation between miR-29b-3p and DNMTs in cardiomyocytes and support the epigenetic normalization of miRNA-based therapy in cardiomyocytes.

## Introduction

Dynamic DNA methylation orchestrates cardiomyocyte development, postnatal maturation and cardiovascular diseases (CVDs) (Gilsbach, et al., 2014). Various studies have suggested that DNA methylation aberrations contribute to the development of CVDs, such as congenital heart disease (CHD), atherosclerosis, hypertension, and cardiac hypertrophy (Fernández-Sanlés, et al., 2017; Zhong, et al., 2016). The global DNA methylation of heart tissues in patients with tetralogy of Fallot (TOF) is lower than that of control tissues, while the *NKX2.5*, *HAND1*, *RXRA* and *TBX5* promoters were present under high methylation conditions (Sheng, et al., 2013; Sheng, et al., 2014; Sheng, et al., 2012). The global DNA methylation level decreased in patients with ischemic cardiac disease, atherosclerosis or essential hypertension, while the methylation level of the estrogen receptor (ER)-α or 11 β-hydroxysteroid dehydrogenase 2 (*11βHSD2*) promoter increased (Friso, et al., 2008; Huang, et al., 2009; Smolarek, et al., 2010; Ying, 2000). These studies revealed that DNA methylation in the global genome and specific genes present their own specific methylation patterns in CVDs. DNA methyltransferases (*DNMTs*), including *DNMT1*, *DNMT3A* and *DNMT3B*, participate in the process of DNA methylation, where *DNMT1* functions in the maintenance of DNA methylation and *DNMT3A* and *DNMT3B* mediate *de novo* DNA methylation (Rideout, et al., 2001). *DNMT* siRNA disrupted the assembly of sarcomeres and reduced the beating frequency, contraction movement, field action potential amplitude and cytosolic calcium signal of cardiomyocytes (Fang, et al., 2016).

An increasing number of studies have focused on the role of miRNAs, which contain 21–25 nucleotides. They regulate posttranscriptional gene expression through mRNA cleavage and degradation or translational inhibition, which depends on the degree of complementarity between miRNA and target mRNA sequence (Kasinski and Slack 2011). Aberrant miRNA expression patterns have been reported in various CVDs, including cardiac hypertrophy, fibrosis, heart failure, arrhythmia, atherosclerosis and TOF (Bruneau 2008; Liu and Olson 2010). A number of clinical studies have also shown that the miR-29 family plays a role in the occurrence and development of CHD. Among 21 patients with TOF, the expression patterns of 18 miRNAs were significantly different, and the expression of miR-29c, which belongs to the miR-29c family, was downregulated (Zhang, et al., 2013a). Maternal blood tests of 30 CHD fetuses showed that miR-29c is significantly elevated in pregnant women with fetal VSD, ASD, and TOF (Nagy, et al., 2019; Zhu, et al., 2013). In patients with persistent atrial fibrillation (AF) after rheumatic heart disease, miR-29b-1-5p and miR-29b-2-5p interact with 24 downregulated circRNAs to participate in the remodeling of heart structure in patients with AF (Hu, et al., 2019). It has been reported that miRNA expression patterns are often disrupted by aberrant DNA methylation in many diseases. Many miRNAs were downregulated or upregulated by DNA hypermethylation or hypomethylation, respectively. Saito et al*.* (Saito, et al., 2006) first found that miR-127 upregulation was associated with its DNA methylation status. It has been reported that CpG island hypermethylation occurs in the miR-124a (Lujambio, et al., 2007), miR-34 (Chen, et al., 2012; Suzuki, et al., 2010) and miR-9 families (Roman-Gomez, et al., 2009), which is related to the transcriptional inactivation of these miRNAs in human tumors. It has been widely reported that hypermethylation of specific CpG islands in gene promoter regions is a common mechanism of miRNA silencing.

miRNAs are novel regulators of DNA methylation and act by targeting methylation-related proteins, including *DNMTs*, *MBD2*, *MBD4* and *MeCP2* (Wang, et al., 2017). miR-101 inhibited the expression of *DNMT3A*, resulting in a decrease in global DNA methylation in lung cancer (Yan, et al., 2014). The expression of *DNMT3A* and *DNMT3B* was high in lung cancer, and the expression of the miR-29 family was negatively correlated with them. Further study showed that the miR-29 family directly targets the 3′ untranslated regions (3′UTRs) of *DNMT3A* and *DNMT 3B* (Fabbri, et al., 2007). The expression of miR-17–92 was decreased in patients with idiopathic pulmonary fibrosis (IPF), while the expression of *DNMT1* increased. Further study identified that several miRNAs from the miR-17–92 cluster targeted the *DNMT1* gene (Dakhlallah, et al., 2013). It was shown that miR-212 repressed the expression of *MeCP2* in gastric cancer (Wada, et al., 2010) and miR-373 suppressed the expression of *MBD2* in hilar cholangiocarcinoma (Chen, et al., 2011).

miRNAs regulate DNA methylation by modulating methylation-related proteins and expand strategies based on this to treat diseases with aberrant DNA methylation. The miR-29 family, the most widely studied epigenetic factor, reverted aberrant methylation by targeting *DNMT3A* and *DNMT3B* (Garzon, et al., 2009; Morita, et al., 2013; Qiu, et al., 2018; Zhang, et al., 2018b) in leukemia, in porcine early embryo development or in lung cancer cells, while the interaction between miR-29b-3p and *DNMTs* in CHD remains unknown. Our previous results showed that in patients with CHD, *DNMT1*, *DNMT3A* and *DNMT3B* showed a statistically significant negative correlation with miR-29b-3p expression. In this study, it was also revealed that there was a feedback loop between miR-29b-3p and *DNMTs* in cardiomyocytes. It is worth continuing to explore whether upregulation of DNMT by suppressing miR-29b-3p expression is sufficient to induce effective DNA hypermethylation in cardiomyocytes. Therefore, in the present study, we will explore the interaction between miR-29b-3p and DNA methylation and the efficacy of miRNA in the treatment of hypomethylated zebrafish and cardiomyocytes.

## Materials and Methods

### Patients With CHD

Heart tissues were obtained from 17 patients with CHD (mean age: 24.5 ± 22.0 months; 47.1% female and 52.9% male) between 2014 and 2016 from the Children’s Hospital of Fudan University, Shanghai, China. The residual tissues were trimmed from the right ventricular outflow tract (RVOT) during surgery and immediately stored in liquid nitrogen. The Ethics Committee of Children’s Hospital of Fudan University approved this study.

### RNA Extraction and Quantitative RT-PCR Analysis

Total RNA was isolated from frozen heart tissues, cardiomyocytes or zebrafish using TRIzol reagent (Invitrogen, Carlsbad, CA) according to the manufacturer’s protocol. Reverse transcription was conducted with the PrimeScript RT reagent kit (TaKaRa, Shiga, Japan). RNA expression was quantified with SYBR Premix Ex Taq™ (TaKaRa, Shiga, Japan). The primer sequences are listed in Supplementary Table S4. MiRNAs were reverse-transcribed by the miRcute Plus miRNA First-Strand cDNA Synthesis Kit (TIANGEN, Beijing, China) and quantified by the miRcute Plus miRNA qPCR Detection Kit (TIANGEN, Beijing, China) with specific primers (TIANGEN, Beijing, China). The relative miRNA and mRNA quantification were determined using the comparative CT method and were normalized against U6 for miRNA or β-actin for mRNA.

### Bioinformatics Analysis

The prediction of TF binding sites was performed via TFSEARCH (http://www.cbrc.jp/research/db/TFSEARCH.html). The CpG enrichment regions were analyzed, and the BSP primers were designed by the online MethPrimer software (http://www.urogene.org/methprimer/index1.html). The primers for targeted bisulfite sequencing (MethylTarget) were designed using the online primer3 software (http://primer3.ut.ee/). The TargetScan and PicTar algorithms were used to predict the target genes of miR-29b-3p.

### Cell Culture

The HL1 cell line was provided by Professor Duan Ma (Fudan University, Shanghai, China). The HEK293 cell line was purchased from the Cell Bank of the Chinese Academy of Sciences (Shanghai, China). Both cell lines were cultured in Dulbecco’s modified Eagle medium (DMEM, Gibco, Waltham, MA) containing 10% FBS with 1% penicillin-streptomycin. Cells were cultured in a humid environment with 5% CO_2_ and at 37°C.

### Plasmid Constructs

The promoter regions of the hsa-miR-29b-1 and hsa-miR-29b-2 genes were amplified and inserted into the KpnI and SacI sites of the pGL3-promoter vector (Promega, Madison, Wisconsin) to generate the pGL3-hsa-miR-29b-1/2-promoter plasmid. The primers for PCR amplification are listed in Supplementary Table S5. The *DNMT3A* and *DNMT3B* 3′UTRs from human/rat/mouse genomic DNA were cloned into XhoI and NotI sites downstream of Renilla luciferase in the psiCHECK-2 vector (Promega, Madison, Wisconsin), while the firefly luciferase gene was used as an internal control. Mutation of the *DNMT3A* and *DNMT3B* 3′ UTRs was performed using the Fast Mutagenesis System (TransGen Biotech, Beijing, China). The PCR primers are listed in Supplementary Table S6.

### Transfection and Luciferase Assay

HEK293T cells plated in 96-well plates were transfected with 100 ng of pGL3-basic, pGL3-promoter, pGL3-hsa-miR-29b-1/2-promoter (unmethylated), or mpGL3-hsa-miR-29b-1/2-promoter (methylated) plasmid. The pGL3-basic vector without the promoter sequences was used as a negative control. The pRL-TK plasmid (Promega, Madison, Wisconsin) containing the Renilla luciferase gene was cotransfected with the above plasmid to standardize the luciferase activity.

HL1 cells were cotransfected with psiCHECK-2 vector (100 ng) containing the 3′UTR of *DNMT3A* or *DNMT3B* (WT or MUT) and miRNA mimic (20 pmol) in 96-well plates. The four groups were psiCHECK-2-*DNMT3A*/3B-WT + miR-NC mimic, psiCHECK-2-*DNMT3A*/3B-WT + miR-29b-3p mimic, psiCHECK-2-*DNMT3A*/3B-MUT + miR-NC mimic and psiCHECK-2-*DNMT3A*/3B-MUT + miR-29b-3p mimic. All transfections were performed with Lipofectamine 3000 transfection reagent (Invitrogen, Carlsbad, CA).

Luciferase analysis was performed 24 h later by a dual-luciferase reporter assay (Promega, Madison, Wisconsin) according to the manufacturer’s instructions. After lysis in passive lysis buffer at room temperature for 15 min, the relative Renilla luciferase activity of cultured cells was obtained after normalization to firefly luciferase gene activity through reaction with Luciferase Assay Reagent II and Stop & Glo Reagent.

### BSP and Cloning-Based Sequencing

DNA was subjected to bisulfite modification by the EpiTect bisulfite kit (Qiagen, Hilden, Germany) according to the manufacturer’s protocol. The BSP primers for the promoter regions of the miR-29b-3p gene were designed by the online MethPrimer software (http://www.urogene.org/methprimer/) (Supplementary Table S7). The purified PCR products were used to ligate the pMD™18-T vector (TaKaRa, Shiga, Japan) and were then transformed into DH5α competent cells (TIANGEN, Beijing, China). After 12 h of incubation at 37°C, blue/white and ampicillin selection was performed. Ten different positive clones were randomly selected for sequencing. The BSP sequencing data were analyzed by BIQ Analyzer software (Max Planck Institute for Informatics, Saarbrücken, Germany).

### CpG Methyltransferase (M. SssI) Treatment

M. SssI (New England BioLabs, Beverly, MA) was incubated with 1 µg of pGL3-hsa-miR-29b-1/2-promoter plasmid in 20 µl of 1X NEBuffer 2, 10 mM MgCl_2_, 1 mM dithiothreitol, and 160 µM S-adenosylmethionine for 3 h at 37°C.

### Targeted Bisulfite Sequencing

MethylTarget performed by Genesky Biotechnologies Inc. (Shanghai, China) was used to detect the miR-29 methylation density. The primers used for miR-29 are shown in Supplementary Table S8. A detailed description of the MethylTarget assay was reported previously (Zhang, et al., 2020).

### Western Blot Analysis

Total protein was extracted using RIPA lysis buffer (Beyotime, Shanghai, China) and protease inhibitor cocktail (Thermo Fisher Scientific, Waltham, MA). The protein concentration was determined by a Pierce BCA protein assay kit (Thermo Fisher Scientific, Waltham, MA). Anti-*DNMT1*, anti-*DNMT3A*, anti-*DNMT3B*, and anti-β-actin antibodies and HRP-labeled goat anti-rabbit IgG secondary antibodies were purchased from Cell Signaling Technology (Danvers, MA) and Abcam (Cambridge, MA), respectively. ECL reagents (Merck Millipore, Darmstadt, Germany) were used to visualize specific protein bands.

### Cell Proliferation Assay

Cell viability was measured by the CCK-8 assay (Dojindo Laboratories, Kumamoto, Japan). At 24, 48, and 72 h, the CCK-8 solution was prepared with medium to a concentration of 10%, 100 µl of the mixed solution was added to each 96-well plate, and the operation was protected from light. The plates were incubated at 37°C for 2.5 h, and the absorbance at 450 nm was then measured.

### EdU Incorporation Assay

The EdU incorporation assay was performed according to the manufacturer’s protocol (Life Technologies, Waltham, MA). The cell proliferation rate was calculated as the proportion of nucleated cells incorporated into EdU to the total number of cells by randomly selecting 10 high-power fields per well.

### Zebrafish Embryology and Microinjection

Zebrafish breeding, embryo collection and maintenance were carried out in accordance with recognized standard operating procedures. The injection concentration of miRNA inhibitor was 5 μM, and that of 5-azacytidine was 25 µM. At the 1–4 cell stage, 3 nl of miRNA inhibitor or 5-azacytidine was injected into the yolk of each zebrafish embryo. A Leica M205 FA digital camera was used to photograph the embryos, and Adobe Photoshop CS5 software was used to process the digital images.

### 5-Azacytidine and 5-aza-2′-Deoxycytidine (Decitabine) Working Solution

5-azacytidine powder (Sigma-Aldrich, St. Louis, MO) was dissolved in an appropriate amount of DMEM (cardiomyocyte treatment) or blue egg water (zebrafish embryo treatment). The concentration of the stock solution was 500 μM. The working solution was diluted to 25 µM. Five milligrams of decitabine powder (Sigma-Aldrich, St. Louis, MO) was dissolved in 1 ml of DMSO. The working solution was diluted to 20 µM. The stock solutions were stored at −80°C.

### General Morphology Score System

The general morphology score (GMS) system is used as a quantitative assessment method to evaluate the development of zebrafish embryos, which displays the development scores of zebrafish embryos at 24 hpf, 48 hpf and up to 72 hpf, with different scores assigned to specific developmental endpoints (Hermsen, et al., 2011). It included evaluation indicators such as tail detachment, somite formation, eye development, heartbeat and blood flow speed. The full score was 7 at 24 hpf, 12 at 48 hpf and 15 at 72 hpf.

### Shortening Fraction Quantification

The maximum systolic and diastolic frames of the video were saved as JPEGs, and the width of the maximum systolic and diastolic hearts of the ventricles was measured from the image by ImageJ. The ventricular shortening fraction (%) was calculated as follows: ×100 (diastolic width-systolic width)/(diastolic width)%.

### Statistical Analysis

Statistical analysis was carried out by Stata. Values are expressed as the means ± SEM. Spearman’s rank correlation was used to examine the correlation between two continuous variables. One-way analysis of variance (ANOVA) was used to analyze differences among multiple groups. Two-way ANOVA was used to evaluate the expression of miR-29b-3p at different time points after 5-azacytidine or decitabine treatment and the effects of miR-29b-3p and time variables on the proliferation of HL1 cells treated with 5-azacytidine. Student’s t-test was used to determine the statistical significance. Significance was defined as follows: *p < 0.05, ** *p* < 0.01, *** *p* < 0.001, and **** *p* < 0.0001.

## Results

### Negative Correlation Between the Expression of *DNMTs* and miR-29b-3p in Patients With CHD

**FIGURE 1:**
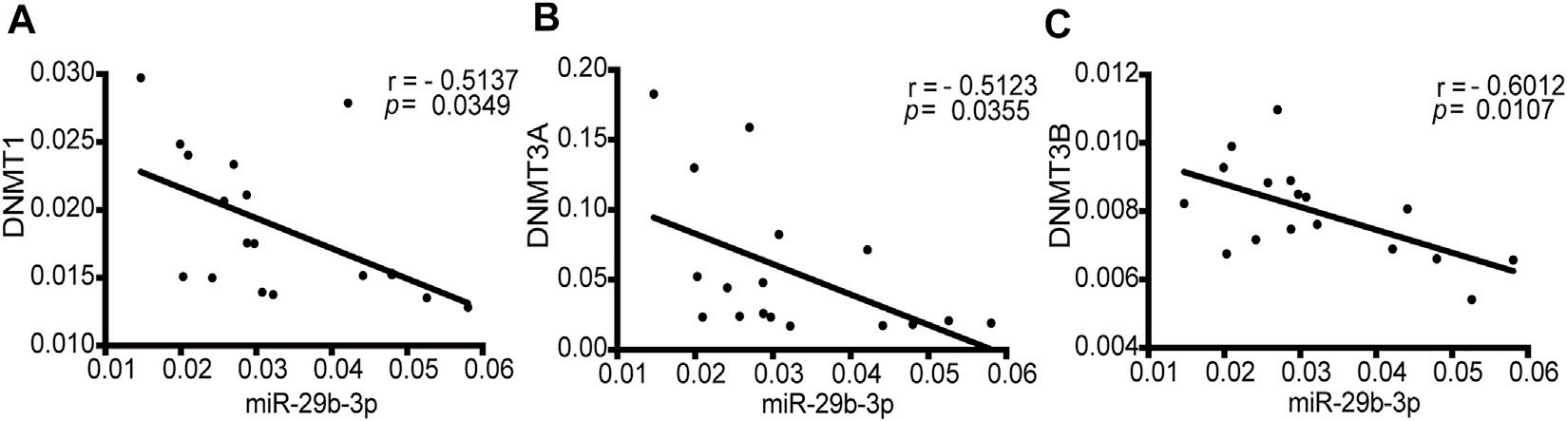
Correlations between the mRNA expression of *DNMTs* and miR-29b-3p in patients with CHD. **(A)** The correlation between *DNMT1* and miR-29b-3p expression (r = −0.5137, *p* = 0.0349). **(B)** The correlation between *DNMT3A* and miR-29b-3p expression (r = −0.5123, *p* = 0.0355). **(C)** The correlation between *DNMT3B* and miR-29b-3p expression (r = −0.6012, *p* = 0.0107). Spearman’s correlation tests were used.

To determine whether a correlation exists between the expression of *DNMTs* and miR-29b-3p in patients with CHD, we analyzed the qPCR data from 17 patients with CHD (Supplementary Tables S1, S2). *DNMT1*, *DNMT3A* and *DNMT3B* showed a statistically significant negative correlation with the expression of miR-29b-3p (r = −0.5137, *p* = 0.0349; r = −0.5123, *p* = 0.0355; and r = −0.6012, *p* = 0.0107, respectively) (Figures 1A–C).

### Transcriptional Regulatory Activity of the miR-29b-1 and miR-29b-2 Gene Promoters

**FIGURE 2:**
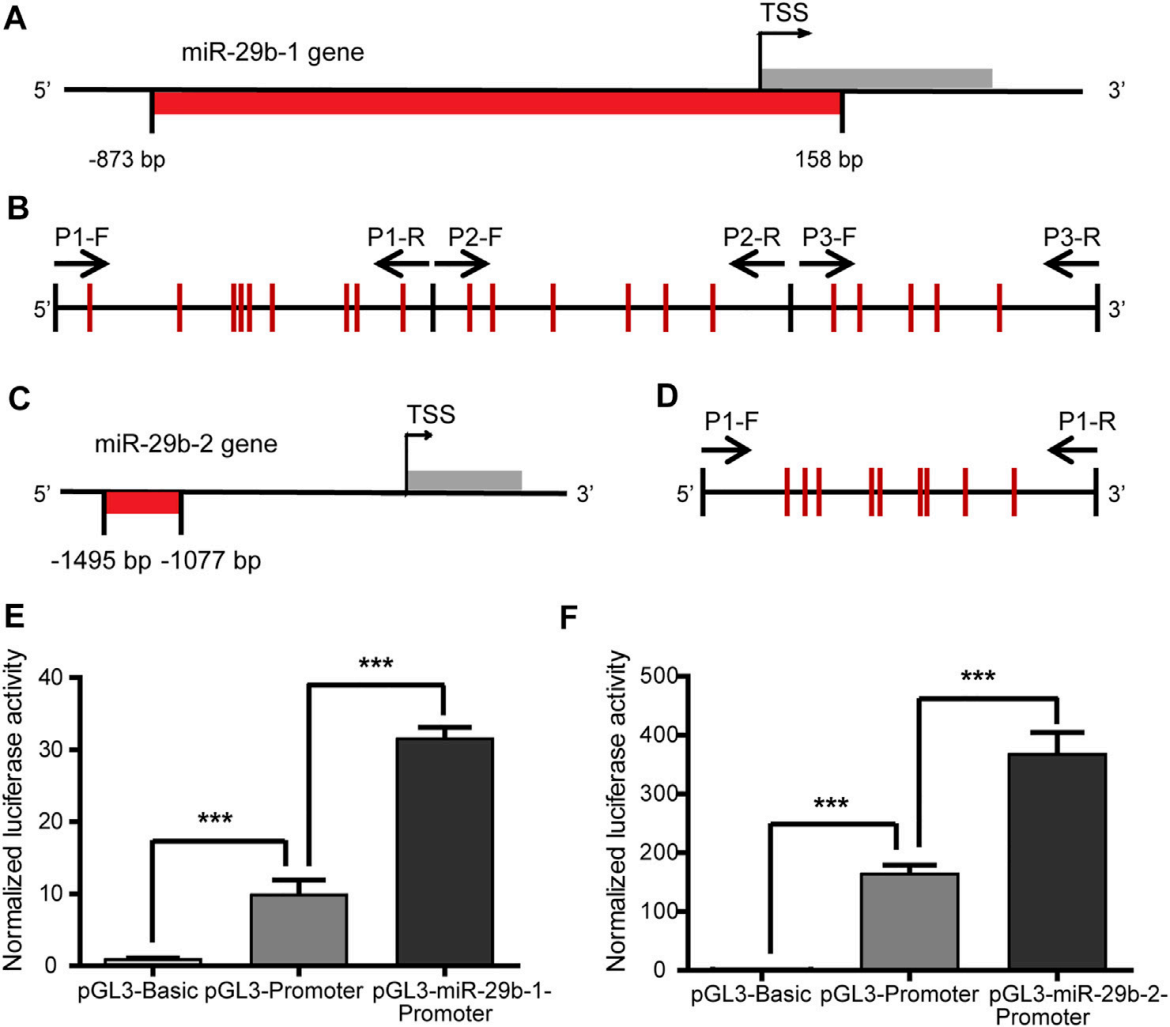
Transcriptional regulatory activity of the miR-29b-1 and miR-29b-2 gene promoters. **(A)** The location of the CpG sites in the promoter region of the miR-29b-1 gene (−873 bp to +158 bp). **(B)** The distribution of the CpG sites and three pairs of primers designed for bisulfite sequencing PCR in the promoter region of the miR-29b-1 gene. **(C)** The location of the CpG sites in the promoter region of the miR-29b-2 gene (−1495 bp to −1077 bp). **(D)** The distribution of the CpG sites and the primers designed for bisulfite sequencing PCR in the promoter region of the miR-29b-2 gene. **(E)** The effect of the −873 bp to +158 bp region on the regulation of miR-29b-1 gene promoter transcriptional activity. **(F)** The effect of the −1407 bp to -1173 bp region on the regulation of miR-29b-2 gene promoter transcriptional activity (***p < 0.001).

As there was a negative correlation between *DNMT* and miR-29b-3p expression in patients with CHD, we further performed experiments to explore whether mutual regulation existed between them. We first detected the DNA methylation status of the miR-29b gene promoter and analyzed its correlation to miR-29b-3p expression in patients with CHD. Hsa-miR-29b-3p is the mature form of premiR-29b-1 and premiR-29b-2. The gene encoding premiR-29b-1 is located on Chr. 7q32.3, while the gene encoding premiR-29b-2 is located on Chr. 1q32.2. A fragment from −1,530 bp to +165 bp relative to the transcription start site (TSS) of the miR-29b-1 gene was shown to have promoter activity and include binding sites for transcription factors Gli, Myc and NF-κB (Mott, et al., 2010). Based on the information analyzed by MethPrimer software, we found a CpG-enriched area from −873 bp to +158 bp relative to the TSS of the miR-29b-1 gene, which contained 20 CpG units (Figures 2A,B, Supplementary Figure S1A). The promoter region of the miR-29b-2 gene has rarely been reported, so we focused on the fragment from −2000 bp to +200 bp relative to the TSS of the miR-29b-2 gene. With the online MethPrimer software, we found a CpG-enriched area containing 9 CpG units, which was located in the −1495 bp to −1077 bp region relative to the TSS of the miR-29b-2 gene (Figures 2C,D, Supplementary Figure S1B).

To identify the transcriptional regulatory activity of the two fragments, the −873 bp to +158 bp region of the miR-29b-1 gene and the −1495 bp to −1077 bp region of the miR-29b-2 gene were cloned into the pGL3-promoter plasmid. The relative luciferase activities of the pGL3-miR-29b-1-promoter and pGL3-miR-29b-2-promoter increased by 3.1 times and 2.2 times, respectively, compared with that of the pGL3-promoter (Figures 2E,F). The pGL3-basic plasmid was the negative control.

### Negative Correlation Between miR-29b-3p Expression and its Promoter Methylation Status in Patients With CHD

**FIGURE 3:**
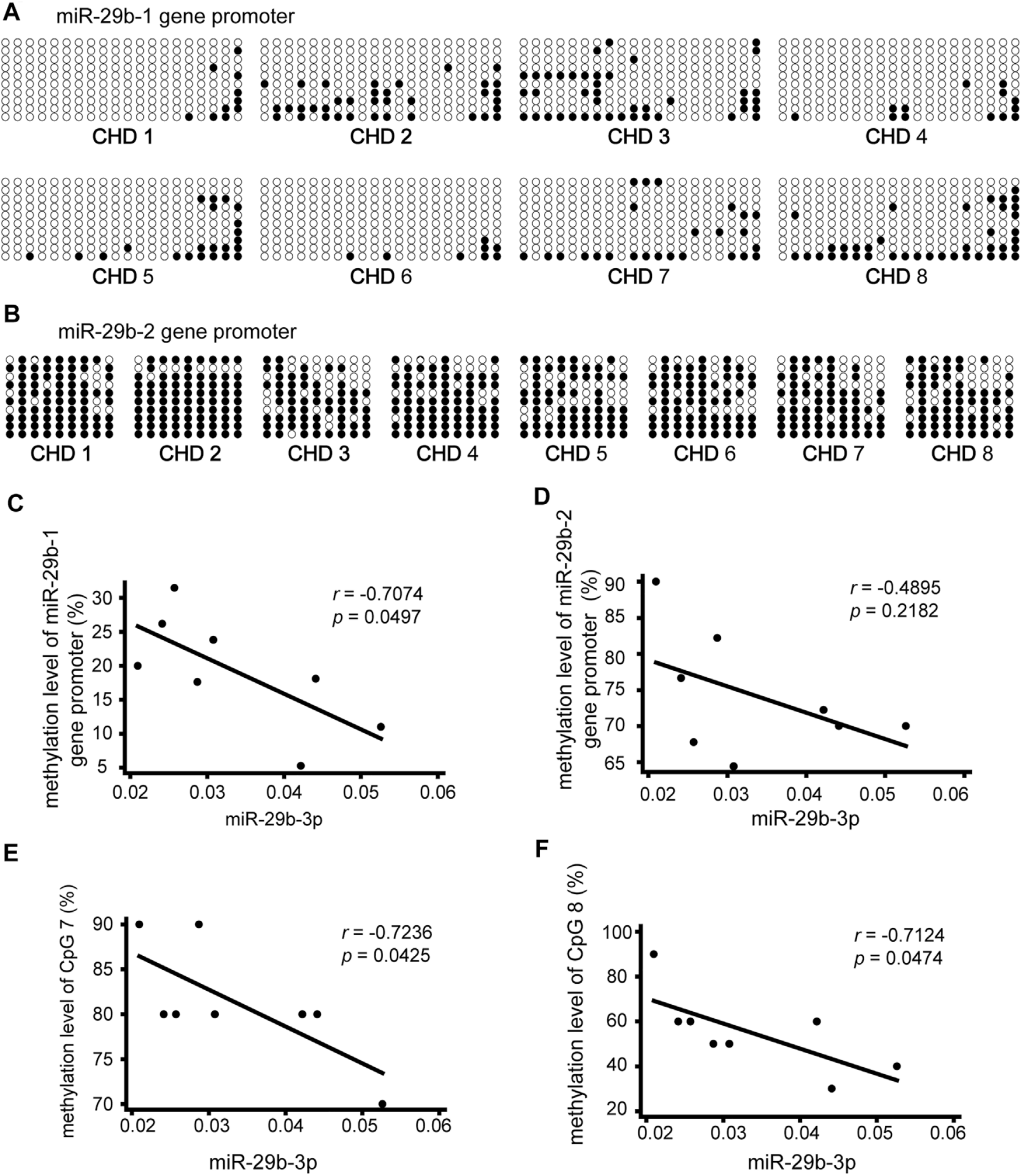
Association of miR-29b-3p expression with its methylation status in eight patients with CHD. **(A** and **B)** Methylation status of the promoter region of the miR-29b-1 gene and miR-29b-2 gene. The black and white circles represent methylated and unmethylated CpG dinucleotides, respectively. **(C)** Correlations between miR-29b-3p expression and the methylation status of the miR-29b-1 gene (r = −0.7074, *p* = 0.0497, and N = 8). **(D)** Correlations between miR-29b-3p expression and the methylation status of the miR-29b-2 gene (r = −0.4895, *p* = 0.2182, and N = 8). **(E)** Correlations between miR-29b-3p expression and the methylation status of CpG 7 located in the miR-29b-2 gene (r = −0.7236, *p* = 0.0425, and N = 8). **(F)** Correlations between miR-29b-3p expression and the methylation status of CpG 8 located in the miR-29b-2 gene (r = −0.7124, *p* = 0.0474, and N = 8). Spearman’s correlation tests were used.

To explore the relationship between miR-29b-3p expression and its promoter methylation status in patients with CHD, we performed Spearman’s correlation tests by Stata. The methylation status of the promoter regions of the miR-29b-1 gene and miR-29b-2 gene was measured in heart tissues obtained from eight patients with CHD by bisulfite sequencing PCR (BSP) sequencing (Figures 3A,B). Three pairs of primers were designed to measure the methylation levels of the miR-29b-1 promoter (P1: −597 bp  ∼  −257 bp, P2: −226 bp  ∼  +108 bp and P3: +103 bp  ∼  +403 bp) (Figure 2B), and 1 pair of primers was designed to measure the methylation levels of the miR-29b-2 promoter (P1: −1407 bp  ∼  −1173 bp) (Figure 2D).

As shown in Figure 3C a significant negative correlation was observed between miR-29b-3p expression and the methylation status of the miR-29b-1 gene (r = −0.7074, *p* = 0.0497, and N = 8). The association between miR-29b-3p expression and the methylation status of the miR-29b-2 gene was negative but not significant (r = −0.4895, *p* = 0.2182, and N = 8) (Figure 3D).

We further analyzed the association between miR-29b-3p expression and the methylation status of each CpG unit. No significant correlations were observed between miR-29b-3p expression and the methylation status of each CpG site located in the miR-29b-1 gene. The associations between miR-29b-3p expression and the methylation status of CpG 7 and CpG eight located in the miR-29b-2 gene were statistically significant (r = −0.7236, *p* = 0.0425; r = −0.7124, *p* = 0.0474) (Figures 3E,F).

### Promoter Hypermethylation Decreased the Expression of miR-29b-3p

**FIGURE 4:**
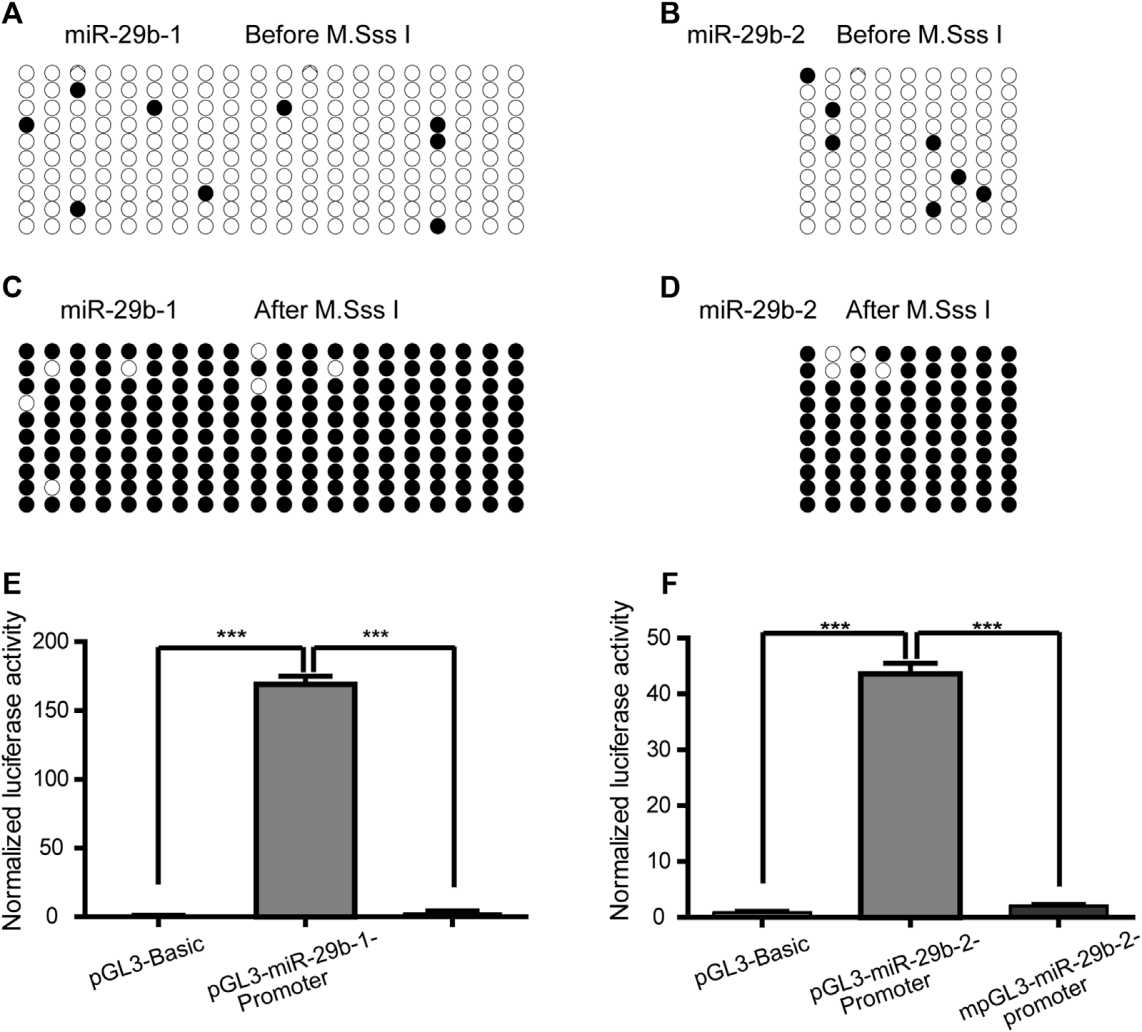
The effect of promoter hypermethylation on the expression of miR-29b-3p. **(A** and **B)** The methylation levels of the pGL3-miR-29b-1 promoter and pGL3-miR-29b-2 promoter before M. SssI treatment. For each plasmid, the methylation status of CpG units is shown for ten clones. Black and white circles indicate the methylated and unmethylated CpG units, respectively. **(C** and **D)** The methylation levels of the pGL3-miR-29b-1 promoter and pGL3-miR-29b-2 promoter after M. SssI treatment. **(E)** The effect of methylation on the transcriptional activity of the −873 bp to +158 bp region of the miR-29b-1 gene. **(F)** The effect of methylation on the transcriptional activity of the −1407 bp to −1173 bp region of the miR-29b-2 gene. (^ns^ not significant, *p < 0.05, **p < 0.01, ***p < 0.001, and ****p < 0.0001).

To explore the impact of methylation on the transcriptional regulatory activity of the two fragments in the miR-29b-1 and miR-29b-2 gene promoters, the pGL3-miR-29b-1-promoter plasmid and pGL3-miR-29b-2-promoter plasmid were methylated by M. SssI methylase. Before M. SssI treatment, the total methylation levels of the 20 inserted CpG units and 9 CpG units were 4.5 and 7.8%, respectively (Figures 4A,B). After M. SssI treatment, the overall methylation status was 96.5 and 95.5%, respectively (Figures 4C,D). Methylated pGL3-miR-29b-1/2-promoter plasmids (mpGL3-miR-29b-1/2-promoter) or unmethylated pGL3-miR-29b-1/2-promoter plasmids were then transfected into HEK293T cells and assayed for dual-luciferase activities. The relative luciferase activity of the mpGL3-miR-29b-1 promoter decreased 58-fold compared with that of the pGL3-miR-29b-1 promoter, while the relative luciferase activity of the mpGL3-miR-29b-2 promoter decreased nearly 20-fold compared with that of the pGL3-miR-29b-2 promoter (Figures 4E,F).

### Gene Hypomethylation Increased the Expression of miR-29b-3p

**FIGURE 5:**
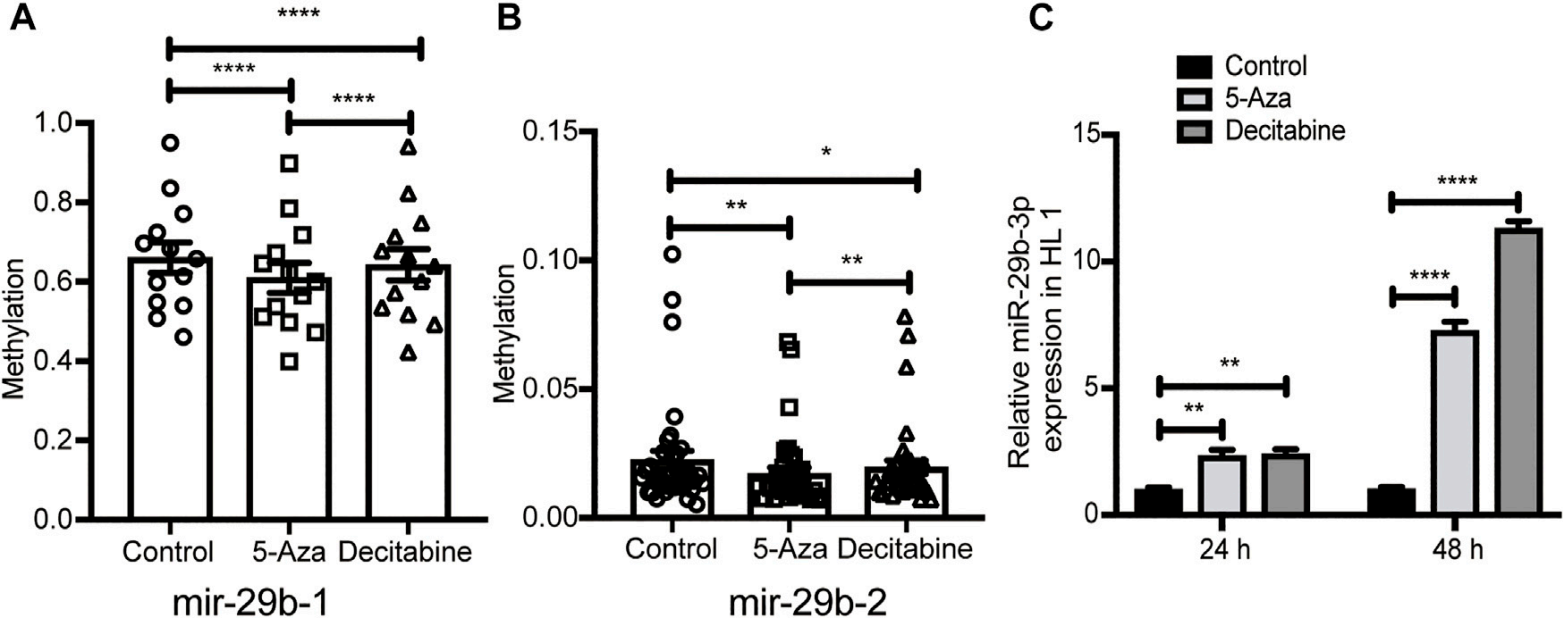
Identification of miR-29b methylation level and mRNA expression after exposure to 5-azacytidine or decitabine. **(A** and **B)** The methylation level of miR-29b-1 and miR-29b-2 in additional samples from the control (n = 3), 5-Aza (n = 5) and decitabine (n = 3) groups analyzed by targeted bisulfite sequencing. **(C)** miR-29b-3p expression in HL1 cells after exposure to 5-azacytidine at 25 µM or decitabine at 20 µM. *p*-values were calculated by one-way ANOVA. ^ns^ not significant; *p < 0.05; **p < 0.01; ***p < 0.001; and ****p < 0.0001.

To explore the role of hypomethylation in the regulation of miR-29b-3p expression *in vitro*, we treated HL1 cells with 5-azacytidine or decitabine, which functions as a potent DNA methyltransferase inhibitor. The results showed that the mir-29b gene was hypomethylated in HL1 cells treated with 5-azacytidine or decitabine, as analyzed by the MethylTarget assay (Figures 5A,B). Moreover, the expression of miR-29b-3p in the 5-azacytidine or decitabine-treated groups was higher than that in the control group at different time points, and the highest expression level was detected at 48 h (Figure 5C).

### miR-29b-3p Directly Targeted the 3′UTRs of *DNMT3A* and *DNMT3B*

**FIGURE 6:**
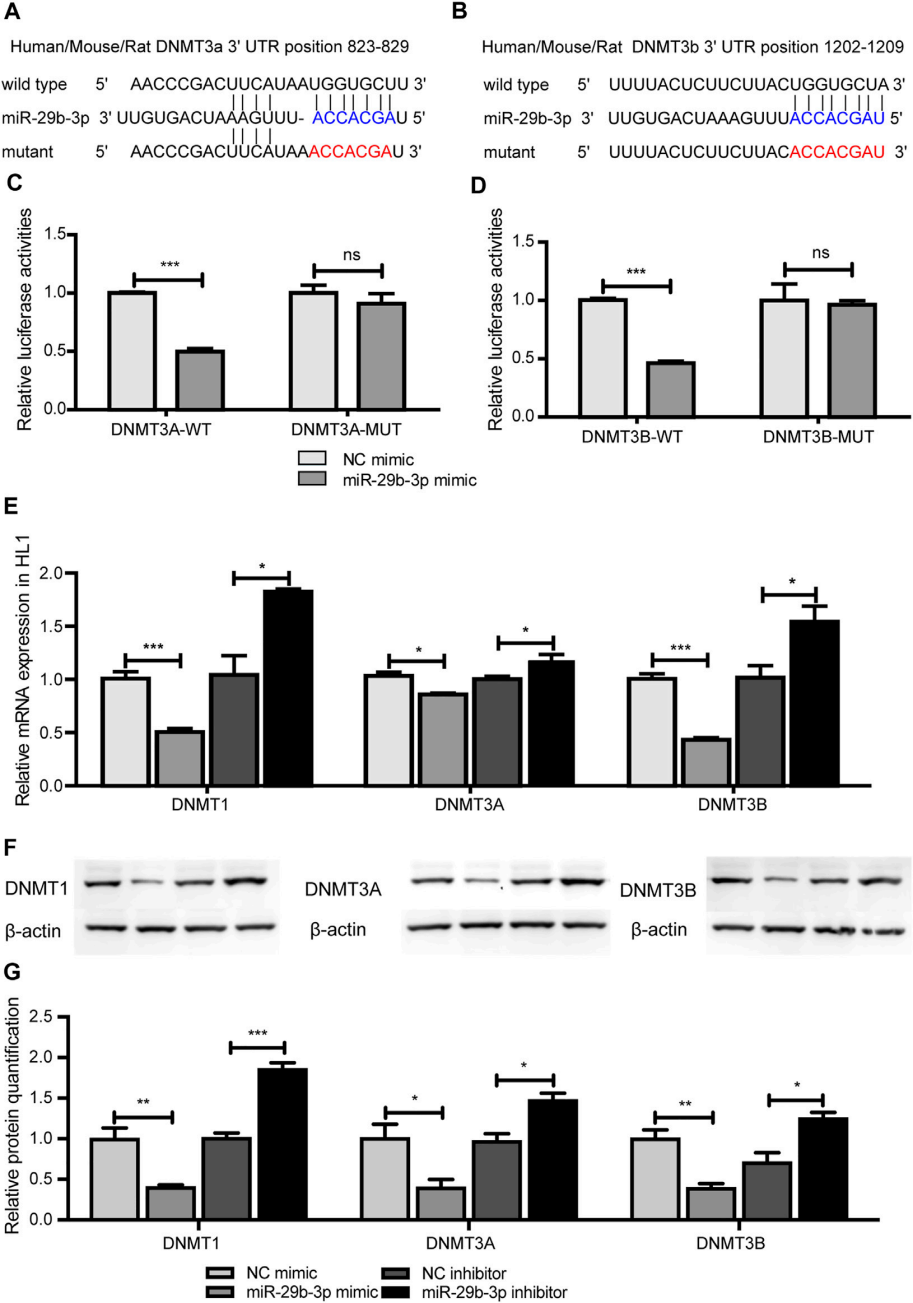
The effect of miR-29b-3p on the expression of *DNMTs*. **(A** and **B)** The predicted binding site of miR-29b-3p in the 3′ untranslated regions of *DNMT3A* and *DNMT3B*. **(C** and **D)** The relative luciferase activity of HL1 cells cotransfected with miR-29b-3p mimic or NC mimic and plasmid containing DNMT3A or 3B wild-type or mutated 3′UTRs. (miR-29b-3p mimic + psiCHECK™-2-DNMT3A/3B-3′UTR vs. NC mimic + psiCHECK™-2-DNMT3A/3B-3′UTR, ***p < 0.001; miR-29b-3p mimic + psiCHECK™-2-DNMT3A/3B-3′UTR-MUT vs. NC mimic + psiCHECK™-2-DNMT3A/3B-3′UTR-MUT, *p* = ns). **(E)** mRNA expression of *DNMTs* in HL1 cells transfected with miR-29b-3p mimic or its inhibitor (miR-29b-3p mimic vs. NC, *p < 0.05, miR-29b-3p inhibitor vs. NC inhibitor, ***p < 0.001). **(F)** Protein expression of *DNMTs* in HL1 cells transfected with miR-29b-3p mimic or its inhibitor. **(G)** Relative quantification of *DNMT* proteins (^ns^ not significant, *p < 0.05, **p < 0.01 and ***p < 0.001).

We identified that DNA methylation regulated the expression of miR-29b-3p, and further study aimed to explore the regulatory effect of miR-29b-3p on the expression of *DNMTs*. miR-29b-3p and *DNMTs* are highly conserved in humans, rats, mice and zebrafish (Supplementary Figure S2). We used bioinformatic tools (PicTar and TargetScan algorithms) to predict the targets of miR-29b-3p, and *DNMT3A* and *3B* were the putative targets of miR-29b-3p, while the 3′UTR of *DNMT1* showed no complementary binding site with miR-29b-3p (Figures 6A,B).

To ascertain the function of miR-29b-3p on *DNMT3A* and *DNMT3B*, a fragment containing the 3′UTRs of *DNMT3A* of *DNMT3B* was spliced to the 3′-end of the synthetic Renilla luciferase reporter gene in the psiCHECK™-2 vector. HL1 cells were cotransfected with miR-29b-3p mimic and psiCHECK™-2-*DNMT3A*-3′UTR plasmid or psiCHECK™-2-*DNMT3B*-3′UTR plasmid and cultured for 48 h. The results showed that miR-29b-3p mimic inhibited the relative luciferase activity of the psiCHECK™-2-*DNMT3A*-3′UTR plasmid and psiCHECK™-2-*DNMT3B*-3′UTR plasmid. We further mutated the 3′UTRs of *DNMT3A* and *DNMT3B* and cotransfected them with miR-29b-3p mimic into HL1 cells. The inhibitory effect of the miR-29b-3p mimic on relative luciferase activity was abrogated after cotransfection with the psiCHECK™-2-*DNMT3A*-3′UTR-MUT plasmid and psiCHECK™-2-*DNMT3B*-3′UTR-MUT plasmid (Figures 6C,D). The mRNA expression of *DNMTs* in HL1 cells transfected with the miR-29b-3p mimic decreased, while the expression increased in the miR-29b-3p inhibitor group (Figure 6E). Furthermore, the protein expression of *DNMTs* was significantly altered by the miR-29b-3p mimic and its inhibitor (Figures 6F,G).

### miR-29b-3p Inhibitor Relieved the Aberration of Zebrafish Embryos Treated With 5-azacytidine

On the one hand, miR-29b-3p regulates DNA methylation by targeting *DNMTs*; on the other hand, the miR-29b gene promoter can be hypermethylated or hypomethylated due to its transcriptional ability. Understanding the crosstalk between miR-29b-3p and DNA methylation may promote the discovery of novel therapeutic targets.

**FIGURE 7:**
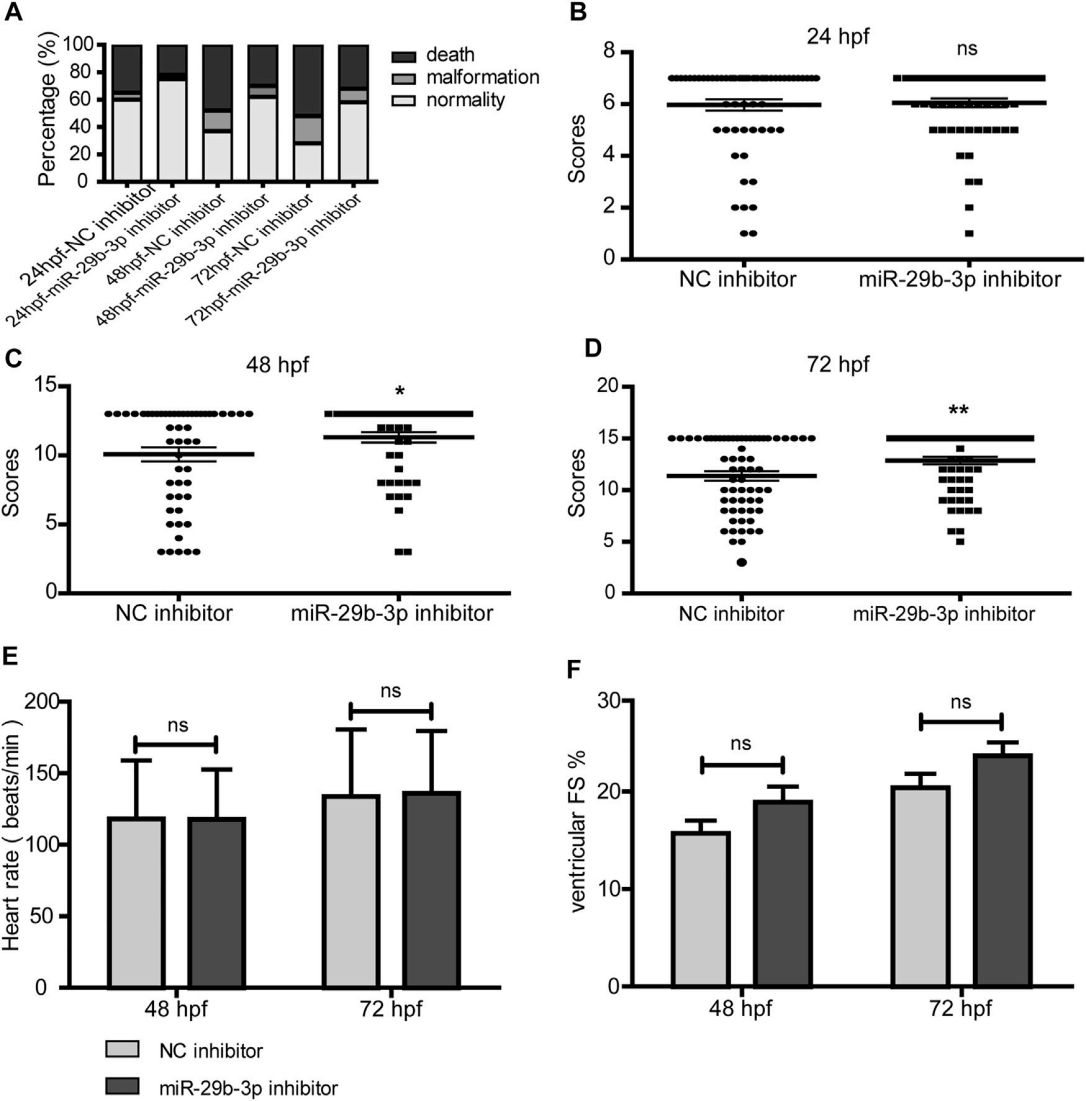
The impact of miR-29b-3p inhibitor on the overall development of hypomethylated zebrafish embryos. **(A)** miR-29b-3p inhibitor partially reduced the mortality and deformity rates of zebrafish that resulted from 5-azacytidine exposure (n > 100). **(B–D)** The zebrafish general development score of the miR-29b-3p inhibitor group was significantly better than that of the NC inhibitor group at 48 hpf and 72 hpf (n > 50). **(E** and **F)** The heart rate and fractional shortening of zebrafish embryos coinjected with 5-azacytidine and miR-29b-3p inhibitor displayed no significant difference from those of the 5-azacytidine and NC inhibitor groups (n = 20).

To evaluate the impact of the miR-29b-3p inhibitor on the overall development of hypomethylated zebrafish embryos, we assessed their survival, malformation rate and general morphology score by coinjecting 5-azacytidine and miR-29b-3p inhibitor into the yolk of zebrafish embryos at the 1–4-cell stage. The miR-29b-3p expression in zebrafish embryos injected with 5-azacytidine and/or miR-29b-3p inhibitor is shown in Supplementary Figure S3A. The mRNA and protein expression of *DNMTs* in zebrafish injected with 5-azacytidine and/or miR-29b-3p inhibitor is shown in Supplementary Figures S3B–D. The results showed that 5-azacytidine exposure led to an increased mortality rate and deformity rate in a time-dependent manner, while coinjection with the miR-29b-3p inhibitor partially reduced the mortality and deformity rates (Figure 7D). General development assessed by the GMS system was significantly delayed by 5-azacytidine exposure (Yang, et al., 2019). The miR-29b-3p inhibitor partially promoted the developmental status at 48 h postfertilization (hpf) and 72 hpf (Figures 7G–I). Zebrafish embryos coinjected with 5-azacytidine and NC inhibitor exhibited obvious deformities, including body curvature, yolk sac edema and blood congestion at the cardiac inflow tract at 48 hpf, while coinjection with miR-29b-3p inhibitor relieved the degree of deformity. Representative images of the overview are shown in Supplementary Figure S4.

5-Azacytidine exposure induced a decrease in heart rate and fractional shortening (Yang, et al., 2019). The heart rate of zebrafish embryos coinjected with miR-29b-3p inhibitor displayed no significant difference from that of the control group (Figure 7E). The fractional shortening was 15.7 ± 5.8% at 48 hpf and 20.4 ± 6.5% at 72 hpf in the NC inhibitor group, which showed no significant differences from those of the miR-29b-3p inhibitor group (18.9 ± 7.3% at 48 hpf and 23.7 ± 6.2% at 72 hpf) (Figure 7F).

### miR-29b-3p Inhibitor Increased the Proliferation of Hypomethylated Cardiomyocytes

**FIGURE 8:**
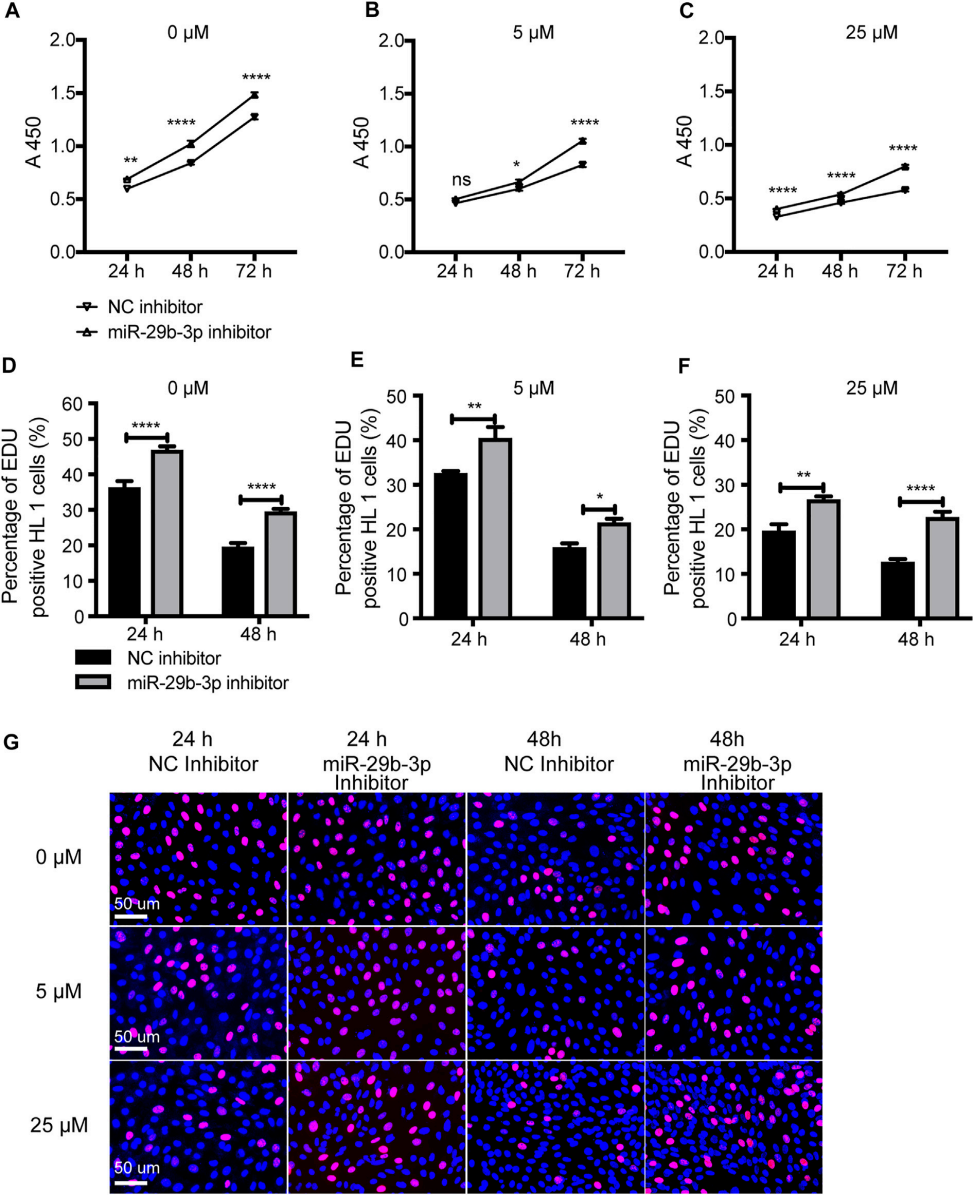
The proliferation ability of hypomethylated cardiomyocytes transfected with miR-29b-3p inhibitor. **(A–C)** Cell proliferation ability detected by a CCK-8 assay at 24, 48 and 72 h **(D–F)** The cell proliferation ability detected by an EdU incorporation assay at 24, 48 and 72 h. **(G)** Representative images of HL1 cells stained with EdU and Hoechst (0 μM, 5 μM, and 25 µM represent 3 concentrations of 5-azacytidine; miR-29b-3p inhibitor vs. NC inhibitor, ^ns^ not significant, *p < 0.05, **p < 0.01, ***p < 0.001, and ****p < 0.0001).

Our previous study showed that a miR-29b-3p inhibitor significantly promoted HL1 cell proliferation (Yang, et al., 2020) (Figures 8A,D). After treatment with 5-azacytidine, the proliferation ability of cardiomyocytes decreased as the concentration increased (Yang, et al., 2019) (Figures 8A–C). Next, we further evaluated the effect of the miR-29b-3p inhibitor on the proliferation of hypomethylated cardiomyocytes *in vitro*. The mRNA and protein expression of *DNMTs* in HL1 cells treated with 5-azacytidine or decitabine or transfected with miR-29b-3p inhibitor is shown in Supplementary Figure S5. The proliferation ability of hypomethylated cardiomyocytes transfected with miR-29b-3p inhibitor or NC inhibitor was detected by CCK-8 and EdU incorporation assays. The proliferation ability of cardiomyocytes exposed to 5-azacytidine at 5 µM was increased after transfection with miR-29b-3p inhibitor compared with that in the control group at 24 h (ns), 48 h (*p* < 0.05) and 72 h (*p* < 0.0001) (Figure 8B). The proliferation ability of cardiomyocytes exposed to 5-azacytidine at 25 µM was significantly increased after transfection with miR-29b-3p inhibitor compared with that in the control group at 24, 48 and 72 h (*p* < 0.0001) (Figure 8C). The EdU assay results also showed that the miR-29b-3p inhibitor promoted hypomethylated HL1 cell proliferation (Figures 8D–F). Representative images of the EdU assay are shown in Figure 8G.

### Effect of miR-29b-3p Inhibitor on the Gene Expression of Hypomethylated Cardiomyocytes

We found that global hypomethylation resulted in increased expression of 2 genes and decreased expression of 20 genes among 45 candidate genes (Yang, et al., 2019). Here, we analyzed the effect of a miR-29b-3p inhibitor on the expression of heart-related genes in hypomethylated cardiomyocytes. The results showed that the expression of *FGF10*, *TNNT2*, *SSB*, *MYH6* and *ERBB3* was decreased in hypomethylated cardiomyocytes (Yang, et al., 2019). was upregulated when transfected with miR-29b-3p inhibitor (Supplementary Table S3).

## Discussion

In this study, we found that the expression of miR-29b-3p was negatively correlated with the expression of *DNMTs* in CHD patients. Further results revealed that there was a feedback loop between miR-29b-3p and *DNMTs* in cardiomyocytes. The reduction in miR-29b-3p expression alleviated the deformity of hypomethylated zebrafish and increased the proliferation and renormalization of gene expression by activating DNMT-dependent DNA methylation in cardiomyocytes.

The miR-29 family is a classic effector of epi-miRNAs, which regulate DNA methylation and demethylation (Garzon, et al., 2009; Zhang, et al., 2013b). In our experiments, the expression of *DNMT1* in HL1 cells treated with miR-29b-3p mimic were downregulated, similar to the results found for *DNMT3A* and *DNMT3B*. The results were consistent with those observed in K562, MV4-11, and Kasumi-1 cells (Garzon, et al., 2009) and in the GC-1 germ cell line (Meunier, et al., 2012). Garzon, R et al. confirmed that miR-29 indirectly downregulates *DNMT1* by directly targeting its transactivator Sp1, a zinc finger transcription factor (Garzon, et al., 2009). Compared with *DNMT3A* and *DNMT3B*, *DNMT1* does not show a complementary binding site with miR-29b-3p and is thus not be directly targeted by miR-29b. Garzon, R et al. confirmed that miR-29 indirectly downregulates *DNMT1* by directly targeting its transactivator Sp1, a zinc finger transcription factor. Our data suggested that miR-29b-3p directly targeted the 3′UTRs of *DNMT3A* and *DNMT3B* while indirectly regulating the expression of *DNMT1*.

Promoter hypermethylation is usually associated with gene silencing, and the higher the methylation of the gene promoter is, the lower the gene expression. Our study suggested that miR-29b-3p expression was negatively related to the methylation status of CpG 7 and CpG 8 located in the miR-29b-2 gene. Several key transcription factors (TFs), including *C/EBP*, *SRF*, *Nrf2* and *HES-1*, were predicted to bind to CpG 7 and CpG 8. The hypermethylation of CpG 7 and CpG 8 may block the binding of TFs to the promoter of the miR-29b-2 gene, resulting in reduced expression of miR-29b-3p. Aberrant DNA methylation may also disrupt the expression of TFs that are essential to the transcription of miRNA, indirectly leading to decreased expression of miRNA. Altered methylation of miRNA-encoding genes may also contribute to aberrant miRNA expression.

The negative correlation between miR-29s and *DNMTs* has been explored in many diseases, such as cholangiocarcinoma (Cao, et al., 2021), osteoarthritis (Dou, et al., 2020), and leukemia (Qiu, et al., 2018). The enforced expression of miR-29s regulated downstream genes mediated by *DNMT3B* (Cao, et al., 2021; Qiu, et al., 2018). *DNMT3B* regulates the miR-29b/PTHLH/CDK4/RUNX2 axis by inducing hypermethylation of specific CpG sites in the miR-29b promoter region, preventing chondrocyte loss due to osteoarthritis (Dou, et al., 2020). Similar to the “DNMT-miR-29” epigenetic circuit, negative feedback regulatory loops between “DNMT1-miR-148/152” in esophageal squamous cell carcinoma and “DNMT1-miR-126” in breast cancer have been reported (Liu, et al., 2015; Zhao, et al., 2011). The feedback loop between miR-29b-3p and *DNMTs* represents a new level of complexity in gene regulation. Exogenous miR-29b-3p inhibitor increased the expression of *DNMTs*, which in turn resulted in a decreased expression of endogenous miR-29b-3p. In our study, an exogenous miR-29b-3p inhibitor relieved the degree of demethylated zebrafish deformity, including body curvature, yolk sac edema and blood congestion at the cardiac inflow tract. Furthermore, the miR-29b-3p inhibitor promoted the proliferation and renormalized the gene expression of hypomethylated cardiomyocytes. These findings illustrate the regulatory role of miR-29 in the normalization of disease epigenetics and provide a theoretical basis for the development of miRNA-based therapeutic strategies.

Epigenetic changes are often reversible, which makes miRNAs attractive in the development of new treatment approaches. Many miRNAs act as biomarkers and prognostic factors for diseases, while only a few are available as therapeutic strategies. This phenomenon may occur for several reasons: one of these reasons may be the absence of pathways to certain physiological organs or tissues (Gallas, et al., 2013; Gavrilov and Saltzman 2012; Pereira, et al., 2017). In our study, a miR-29b-3p inhibitor relieved the deformity of demethylated zebrafish but had no significant effect on heart rate or fractional shortening. However, miR-29b-3p inhibitor increased the proliferation of hypomethylated cardiomyocytes, and this finding is consistent with some research results. Ginkgolide B inhibits hypoxic H9c2 cell apoptosis through miR-29-based inhibition (Ren, et al., 2020). In the ischemia/reperfusion (I/R) injury under PM2.5 exposure, the lncRNA PEAMIR can inactivate the PI3K(p85a)/Akt/GSK3b/p53 cascade pathway that mediates inflammation and apoptosis by downregulating miR-29b-3p (Pei, et al., 2020). The lncRNA TUG1 inhibits apoptosis in H9c2 cells treated with LPS by downregulating miR-29b (Zhang, et al., 2018a). The ineffectivity of the treatment on the cardiovascular system of zebrafish may be due to the injection method, which lacks transmission to specific physiological organs and tissues, resulting in insufficient cellular uptake and processing. Therefore, it is necessary to solve the problem of low cell uptake and processing efficiency. In addition, it is worth noting that some research results are contrary to ours and the above-described studies. *In vivo* experiments of rats induced by endotoxin, doxorubicin or ischemia-reperfusion showed that the upregulation of miR-29b can reduce cardiomyocyte apoptosis, whereas the inhibition of miR-29b exerts the opposite effect (Jing, et al., 2018; Li, et al., 2021; Li, et al., 2020). We speculate that the inconsistent results may be due to different cell types, different experimental models, different expression levels of target mRNA, different degree of cell injury, different stages of tissue development and different doses of miRNA mimic or inhibitor.

Several results from the present study could be serve as motivation for future study. First, the correlations between miR-29b-3p expression and the total methylation level of the miR-29b-2 gene or the methylation status of each CpG site located in the miR-29b-1 gene in patients with CHD were not clear in our study. This may be due to the small sample size, which we will expand to further analyze the relationship between miR-29b-3p expression and the total methylation level of the miR-29b-2 gene. Second, several TFs, including *C/EBP*, *SRF*, *Nrf2* and *HES-1*, were predicted to bind to differentially methylated CpG sites located in the miR-29b-2 gene promoter, and further experiments will focus on the specific regulatory mechanism. Third, mutations in any *DNMT* can cause embryonic lethality in mice (Li, et al., 1992; Okano, et al., 1999). To investigate the specific impact of miRNAs on heart development, a heart-targeted miRNA delivery system needs to be developed for in-depth research.

## Conclusion

These results suggest mutual regulation between miR-29b-3p and *DNMTs* in cardiomyocytes and provide evidence that miRNA-based therapy can normalize the epigenome of cardiomyocytes.

## Data Availability

The original contributions presented in the study are included in the article/Supplementary Material, further inquiries can be directed to the corresponding authors.
