# Supplementary material for: miR-29b-3p Inhibitor Alleviates Hypomethylation-Related Aberrations Through a Feedback Loop Between miR-29b-3p and DNA Methylation in Cardiomyocytes
Source: Front Cell Dev Biol. 2022 Apr 11;10:788799. doi: 10.3389/fcell.2022.788799 (PMC9035530; doi:10.3389/fcell.2022.788799)
Supplement: Supplementary file 1 [file DataSheet1.docx]

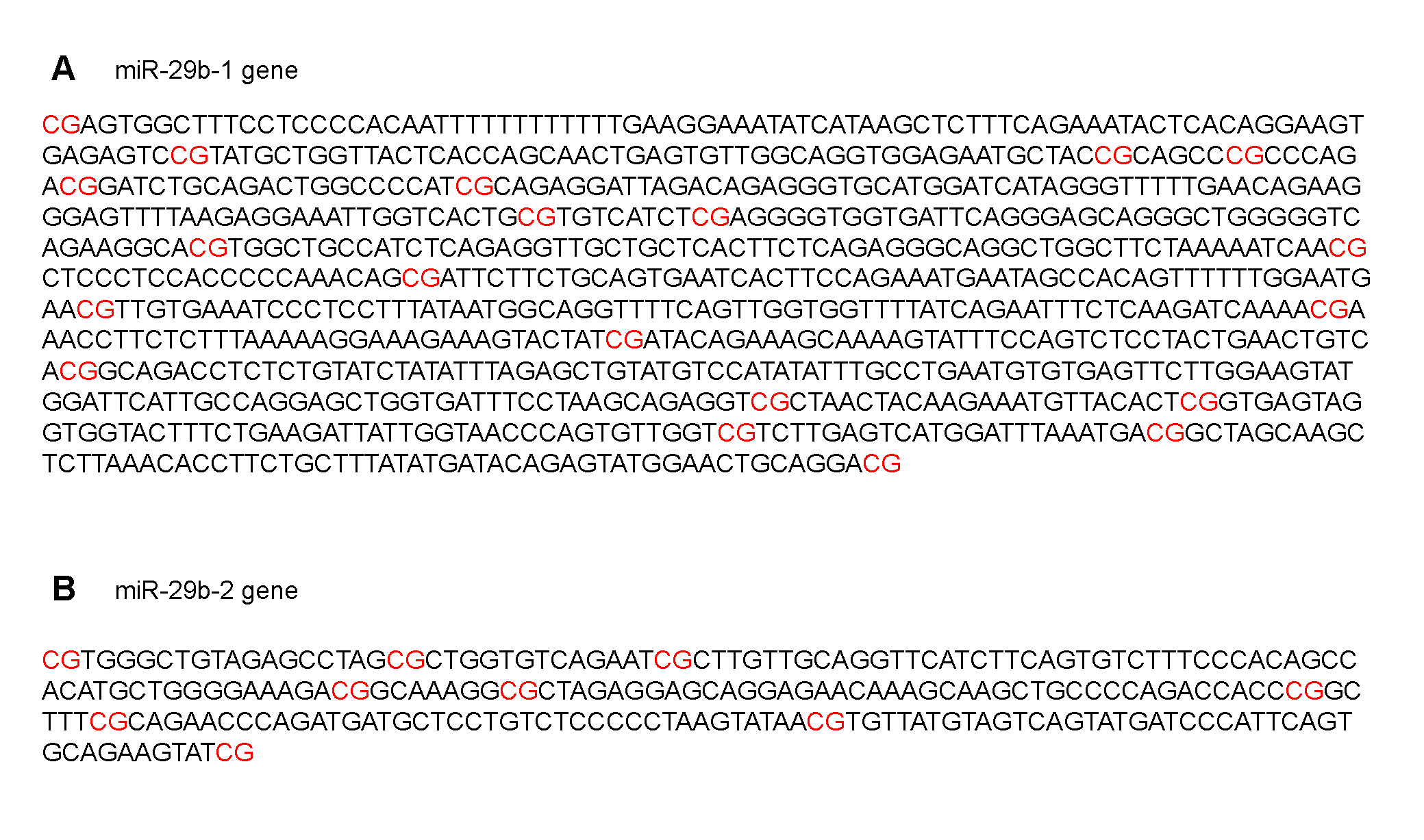


**Figure S1.** Sequences of the promoter regions of the miR-29b-1 and miR-29b-2 genes. A, The sequence represents the 879-base-pair fragment, which contains 20 CpG sites. B, The sequence represents the 234-base-pair fragment, which contains 9 CpG sites.

**
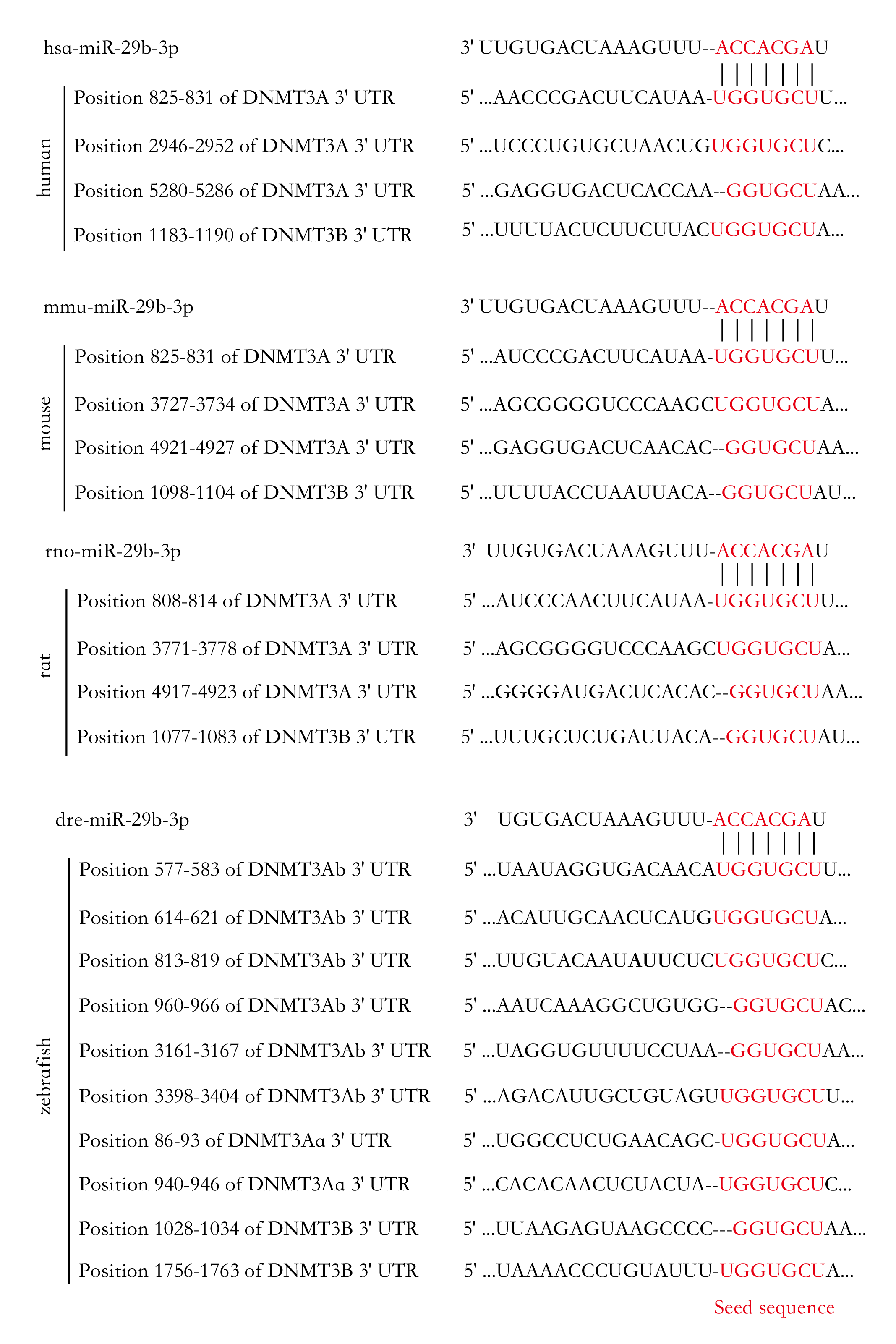
**

**Figure S2.** Comparison of nucleotide sequences of miR-29-3p seed sequences with putative target sequences in different species. The 3′ UTRs of *DNMT3A* and *DNMT3B* contain a putative target site for miR-29-3p that is highly conserved across species.

**
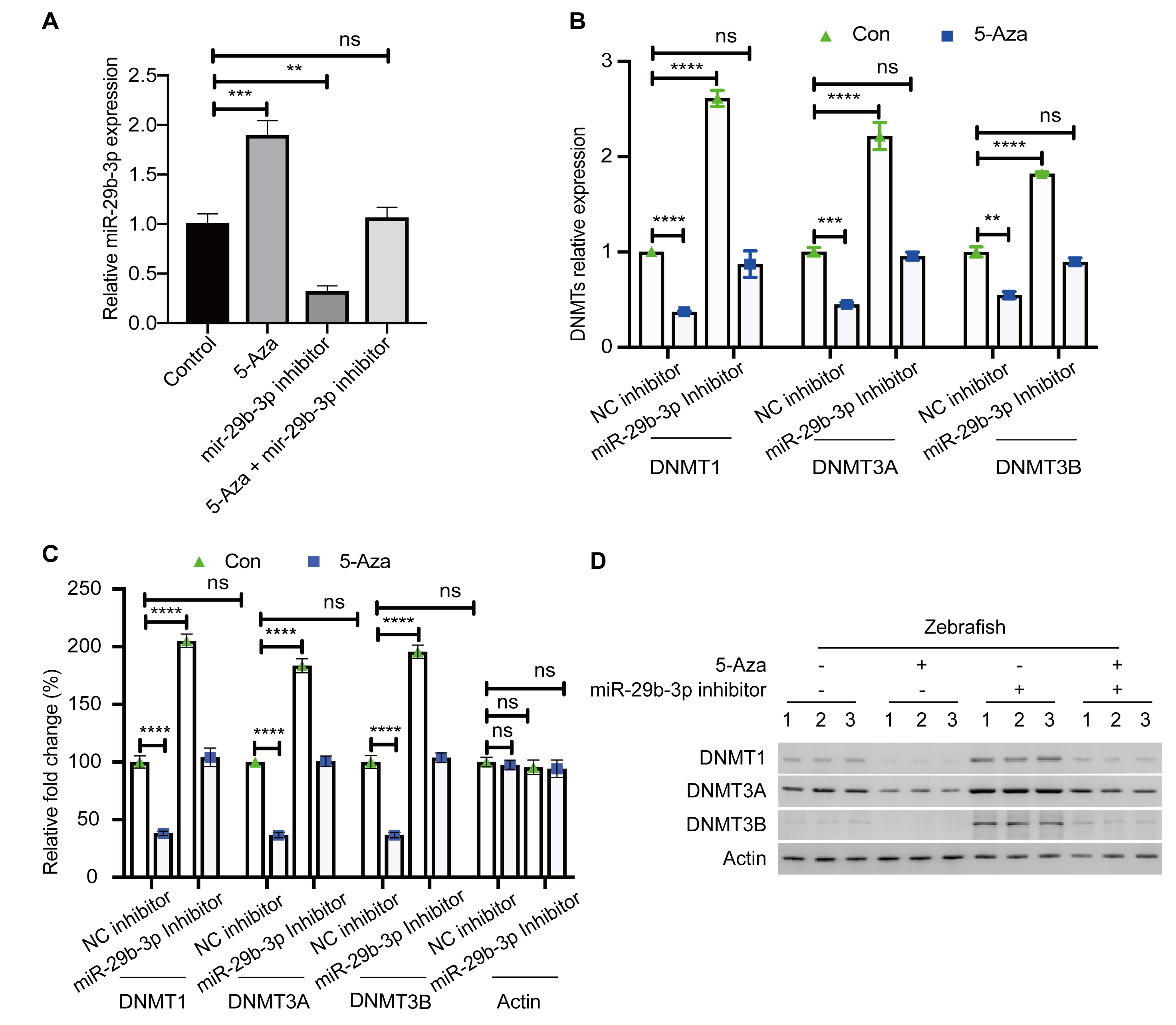
**

**Figure S3.** Expression of miR-29b-3p and the mRNA and protein expression of *DNMTs* in zebrafish embryos at 72 hpf. **A,** The expression of miR-29b-3p was increased in the 5-azacytidine group (***p < 0.001) and decreased in the miR-29b-3p inhibitor group (**p < 0.01). No significant difference was found between the coinjection group and the control group. **B,** DNMT expression was decreased in the 5-azacytidine group (****p < 0.0001) and increased in the miR-29b-3p inhibitor group (****p < 0.0001). No significant difference was found between the coinjection group and the control group. **C-D,** Relative fold change of DNMT proteins expression at 72 hpf in zebrafish embryos. The changing trends were consistent with the mRNA expression results.


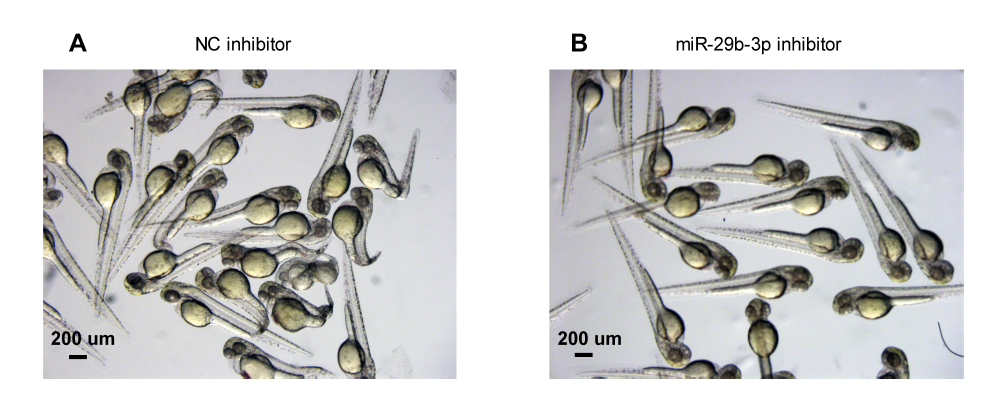


**Figure S4.** Representative images of demethylated zebrafish embryos treated with miRNA inhibitor. A, NC inhibitor. B, miR-29b-3p inhibitor. The arrows indicate deformed zebrafishes.

**
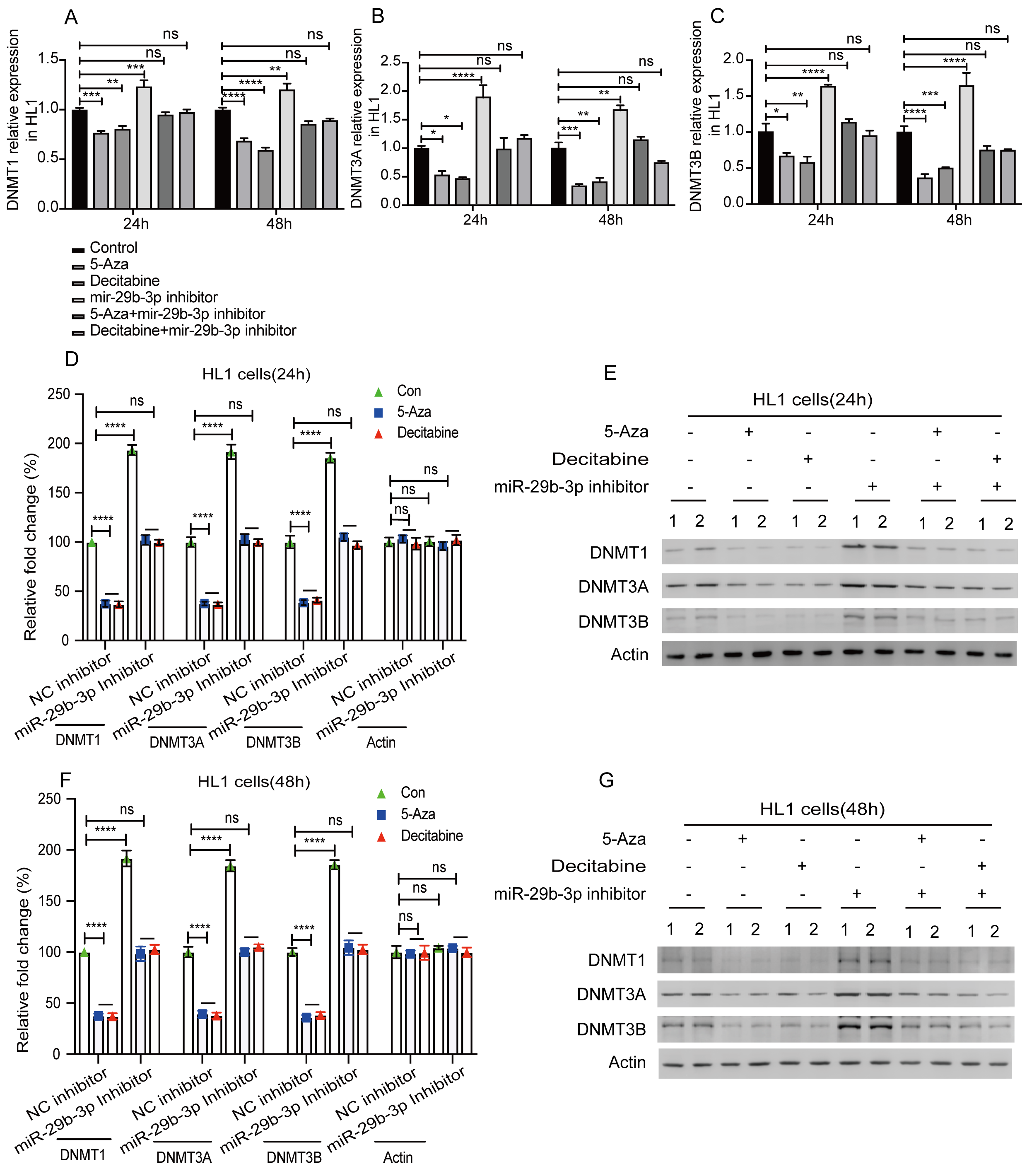
**

**Figure S5.** mRNA and protein expression of *DNMTs* in HL1 cells treated with 5-Aza, decitabine or miR-29b-3p inhibitor. **A-C,** Expression of *DNMT1, DNMT3A,* and *DNMT3B* in HL1 cells at 24 h and 48 h, respectively. The expression was decreased in the 5-azacytidine or decitabine group and increased in the miR-29b-3p inhibitor group. No significant difference was found between the cotreated group and the control group. **D-G,** Relative fold change in DNMT protein expression in HL1 cells at 24 h and 48 h, respectively. Their changing trends were consistent with the mRNA expression results.

**Table S1.** ΔC_t_ and 2^-ΔCt^ values for miR-29b-3p in each CHD

|  |  | miR-29b-3p | | | |
| --- | --- | --- | --- | --- | --- |
| Sample ID | Gender | | Age(months) | ΔC_t_ | 2^-ΔCt^ |
| CHD 1 | female | | 77 | 4.870 | 0.0422 |
| CHD 2 | male | | 36 | 7.090 | 0.0199 |
| CHD 3 | male | | 4 | 6.235 | 0.0257 |
| CHD 4 | female | | 8 | 5.700 | 0.0308 |
| CHD 5 | male | | 6 | 5.895 | 0.0288 |
| CHD 6 | male | | 6 | 7.020 | 0.0203 |
| CHD 7 | male | | 3 | 8.250 | 0.0147 |
| CHD 8 | female | | 48 | 4.565 | 0.0480 |
| CHD 9 | female | | 9 | 6.435 | 0.0241 |
| CHD 10 | female | | 4 | 6.905 | 0.0210 |
| CHD 11 | male | | 12 | 5.800 | 0.0297 |
| CHD 12 | male | | 24 | 6.085 | 0.0270 |
| CHD 13 | male | | 7 | 5.900 | 0.0287 |
| CHD 14 | male | | 24 | 4.760 | 0.0441 |
| CHD 15 | male | | 48 | 4.360 | 0.0526 |
| CHD 16 | female | | 13 | 5.570 | 0.0322 |
| CHD 17 | male | | 7 | 4.150 | 0.0581 |

CHD: congenital heart disease

**Table S2.** ΔC_t_ and 2^-ΔCt^ values for DNMTs in patients with CHD

| Sample ID | DNMT1 | | | DNMT3A | | DNMT3B | |
| --- | --- | --- | --- | --- | --- | --- | --- |
|  | ΔC_t_ | | 2^-ΔCt^ | ΔC_t_ | 2^-ΔCt^ | ΔC_t_ | 2^-ΔCt^ |
| CHD 1 | 6.080 | 0.028 | | 3.745 | 0.071 | 12.040 | 0.0069 |
| CHD 2 | 6.515 | 0.025 | | 2.775 | 0.130 | 10.380 | 0.0093 |
| CHD 3 | 7.015 | 0.020 | | 6.470 | 0.024 | 10.635 | 0.0088 |
| CHD 4 | 8.575 | 0.014 | | 3.485 | 0.082 | 10.900 | 0.0084 |
| CHD 5 | 7.565 | 0.018 | | 6.220 | 0.026 | 11.565 | 0.0075 |
| CHD 6 | 7.920 | 0.015 | | 4.375 | 0.052 | 12.165 | 0.0068 |
| CHD 7 | 5.830 | 0.030 | | 2.340 | 0.183 | 11.025 | 0.0082 |
| CHD 8 | 8.205 | 0.015 | | 7.440 | 0.018 | 12.300 | 0.0066 |
| CHD 9 | 8.235 | 0.015 | | 4.755 | 0.044 | 11.810 | 0.0072 |
| CHD 10 | 6.080 | 0.024 | | 6.550 | 0.023 | 10.050 | 0.0099 |
| CHD 11 | 7.395 | 0.018 | | 6.540 | 0.023 | 10.845 | 0.0085 |
| CHD 12 | 6.440 | 0.023 | | 2.510 | 0.159 | 9.545 | 0.0110 |
| CHD 13 | 6.990 | 0.021 | | 4.565 | 0.048 | 10.600 | 0.0089 |
| CHD 14 | 8.010 | 0.015 | | 7.625 | 0.017 | 11.130 | 0.0081 |
| CHD 15 | 8.465 | 0.014 | | 6.955 | 0.021 | 13.585 | 0.0054 |
| CHD 16 | 8.510 | 0.014 | | 7.700 | 0.017 | 11.460 | 0.0076 |
| CHD 17 | 8.840 | 0.013 | | 7.260 | 0.019 | 12.330 | 0.0066 |

CHD: congenital heart disease

**Table S3**. Effect of miR-29b-3p inhibitor on gene expression of hypomethylated cardiomyocytes

| Genes dysregulated  by 5-azacyditine | 5µM 5-azacyditine  miR-29b-3p inhibitor  vs. NC inhibitor | | 25µM 5-azacyditine  miR-29b-3p inhibitor  vs. NC inhibitor | |
| --- | --- | --- | --- | --- |
|  | Fold  change | *P*  value | Fold  change | *P*  value |
| FGF10 | 1.368↑ | \| 0.000******* \|  \| 0.001674065 \| \| --- \| --- \| --- \| | 1.750↑ | 0.002****** |
| TNNT2 | 1.400↑ | 0.020***** | 1.373↑ | 0.028***** |
| SSB | 1.245↑ | 0.030***** | — | — |
| MYH6 | — | — | 3.095↑ | 0.008****** |
| ERBB3 | — | — | 1.161↑ | 0.032***** |

**P* < 0.05, ***P* < 0.01, ****P* < 0.001, — ns

**Table S4.** Primers used for RT–PCR

| Gene symbol | Sequence | Annealing  temperature |
| --- | --- | --- |
| DNMT1-human | F: CCCCTGAGCCCTACCGAAT  R: CTCGCTGGAGTGGACTTGTG | 60℃ |
| DNMT3A-human | F: GACAAGAATGCCACCAAAGC  R: CGTCTCCGAACCACATGAC | 60℃ |
| DNMT3B-human | F: GAGATTCGCGAGCCCAG  R: TCTCCATTGAGATGCCTGGT | 60℃ |
| β-actin-human | F: TGACGTGGACATCCGCAAAG  R: CTGGAAGGTGGACAGCGAGG | 60℃ |
| DNMT1-mouse | F: AAGAGACGAAAAACGACACG  R: TTAGGGTCGTCTAGGTGCTG | 60℃ |
| DNMT3A-mouse | F: GCCGAATTGTGTCTTGGTGGATGACA  R: CCTGGTGGAATGCACTGCAGAAGGA | 60℃ |
| DNMT3B-mouse | F: ACTTGGTGATTGGTGGAAGC  R: CCAGAAGAATGGACGGTTGT | 60℃ |
| β-actin-mouse | F: GAGACCTTCAACACCCCAGC  R: ATGTCACGCACGATTTCCC | 60℃ |
| DNMT1-zebrafish | F: GGGCTACCAGTGCACCTTTG  R: GATGATAGCTCTGCGTCGAGTC | 60℃ |
| DNMT3A- zebrafish | F: GCTAAGTTTGGTAAAGTGCGG  R: GGATGTCCTCCTTATCATTCA | 60℃ |
| DNMT3B- zebrafish | F: CGTGTTGCCAAGTTCGGG  R: ATCCTCTTTGCCATTCATCA | 60℃ |
| β-actin- zebrafish | F: CGAGCTGTCTTCCCATCCA  R: TCACCAACGTAGCTGTCTTTCTG | 60℃ |
| CTNNB1-mouse | F: GTTCGCCTTCATTATGGACTGCC  R: ATAGCACCCTGTTCCCGCAAAG | 60℃ |
| ERBB3-mouse | F: AGGCTCATTGCTTCTCCTGCCA  R: GAAAATGGGCGCATCGAGCACA | 60℃ |
| FGF10-mouse | F: ATCACCTCCAAGGAGATGTCCG  R: CGGCAACAACTCCGATTTCCAC | 60℃ |
| HEY1-mouse | F: CCAACGACATCGTCCCAGGTTT  R: CTGCTTCTCAAAGGCACTGGGT | 60℃ |
| HOMEZ-mouse | F: GCTGTCCATCAGCCAGATAAGC  R: CAGTGGTTCCATTAGCCAATGCC | 60℃ |
| JAG1-mouse | F: TGCGTGGTCAATGGAGACTCCT  R: TCGCACCGATACCAGTTGTCTC | 60℃ |
| MCTP2-mouse | F: AACGCTTTGTGGAAGACAGCCG  R: GTAGACTCCCATTGGAAGCAGC | 60℃ |
| MED13L-mouse | F: CACGGAGTTTAGGATGGAAGTGG  R: AAGGCTGGAACTGCGGCACTTT | 60℃ |
| MEF2C-mouse | F: CTGAGCGTGCTGTGCGACTGT  R: GCTCTCGTGCGGCTCGTTGTA | 60℃ |
| MYH6-mouse | F: GCTGGAAGATGAGTGCTCAGAG  R: CCAGCCATCTCCTCTGTTAGGT | 60℃ |
| NKX2-2-mouse | F: CAACACGGACCCGCGCTACTC  R: AAGAGCACTCGGCGCTTCCTTC | 60℃ |
| PITX2-mouse | F: AGAAATCGCCGTGTGGACCAAC  R: CCAAAGCCATTCTTGCACAGCTC | 60℃ |
| RAF1-mouse | F: CTTCAGGAACGAGGTGGCTGTT  R: TGCTGCCTTCACACCACTGAGT | 60℃ |
| RHD-mouse | F: CTGCCTTCAGATAGTGACAGAGC  R: TGAGGAGTCCAGTCACCATACC | 60℃ |
| SMAD1-mouse | F: CTGAAGCCTCTGGAATGCTGTG  R: CAGAAGGCTGTGCTGAGGATTG | 60℃ |
| SMYD4-mouse | F: CTGTGCTATGCCAATCGCTCT  R: CACCAGGCATTCTGTCTTACGC | 60℃ |
| SSB-mouse | F: GAGCATGAAGGAAGACACAAGCC  R: GTCTGGTCATCCAAGTCACCTG | 60℃ |
| TBX1-mouse | F: CGAGATGATCGTCACCAAGGCA  R: GTCATCTACGGGCACAAAGTCC | 60℃ |
| TBX2-mouse | F: TCATCGCTGTCACTGCCTACCA  R: CGGCTTACAGTGCTCCTCATAC | 60℃ |
| TNNC1-mouse | F: GATGGTTCGGTGCATGAAGGAC  R: CTTCCGTAATGGTCTCACCTGTG | 60℃ |
| TNNT2-mouse | F: GCTACAGACTCTGATCGAGGCT  R:GCTCATTGCGAATACGCTGCTG | 60℃ |
| ZFPM2-mouse | F:ATGGCAAGGAGTGGAAGACAGC  R:AAGTCCACCACAAAGGCGACGA | 60℃ |

**Table S5.** pGL3-hsa-miR-29b-1/2-promoter plasmid primers

| Gene | Sequence | Length |
| --- | --- | --- |
| miR-29b-1-promoter-F | 5’-AGAGGGTACCCACGTTGCATGCTTTTAGCTTTGC-3’ | 834 bp |
| miR-29b-1-promoter-R | 5’-AGAGGAGCTCCACTGGGTTACCAATAATCTTCAGAAAGTACC-3’ |  |
| miR-29b-2-promoter-F | 5’-AGAGGGTACCGGTGTTGGCTACAGGTGTCATCACTG-3’ | 418 bp |
| miR-29b-2-promoter-R | 5’-AGAGGAGCTCCCACTGCAGCCTGCTCCATAACTT-3’ |  |

**Table S6.** psiCHECK-2-DNMT3A/3B-3’ UTR vector primers

| Primer name | Primer sequence |
| --- | --- |
| psiCHECK2-DNMT3A-WT-3’UTR-F | 5’-AGAGCTCGAGGGGACATGGGGGCAAACTGAG-3’ |
| psiCHECK2-DNMT3A-WT-3’UTR-R | 5’-AGAGGCGGCCGCACAGGAAAGCACCAGTACGTTTTGTATGT-3’ |
| psiCHECK2-DNMT3B-WT-3’UTR-F | 5’-AGAGCTCGAGTTCCAGCCAGGCCCCAAGC-3’ |
| psiCHECK2-DNMT3B-WT-3’UTR-R | 5’-AGAGGCGGCCGCTAGACAAATACTGATTTTAATTAAACATAAGGTAAACTC-3’ |
| psiCHECK2-DNMT3A-MUT-3’UTR-F | 5’-AACCCGACTTCATAAACCACGATTCAAACAGC-3’ |
| psiCHECK2-DNMT3A-MUT-3’UTR-R | 5’-TCGTGGTTTATGAAGTCGGGTTGTACAGTAGT-3’ |
| psiCHECK2-DNMT3B-MUT-3’UTR-F | 5’-TTTTACTCTTCTTACACCACGAATTTTGTAGAATAAGG-3’ |
| psiCHECK2-DNMT3B-MUT-3’UTR-R | 5’-TCGTGGTGTAAGAAGAGTAAAAGGTGTTAAAAACC-3’ |

**Table S7.** Primers for BSP

|  | Primer | Sequence (5’-3’) | Product size(bp) |
| --- | --- | --- | --- |
| miR-29b-1 | P1 | F:TTAGTTTTGTTTGTTGAAGTTGT  R:ACAACCTCTAAAATAACAACCA | 362 |
|  | P2 | F:TTAGTTTTGTTTGTTGAAGTTGT  R:CCAAAAACTCACACATTCAAAC | 334 |
|  | P3 | F:TTTGGAAGTATGGATTTATTGTT  R: TTCCTCCTCCAAAAATCTAATA | 300 |
| miR-29b-2 | P1 | F:GGTGTTGGTTATAGGTG  R:CCACTACAACCTACTCCAT | 418 |

F: forward; R: reverse

**Table S8.** Primers for MethylTarget

|  | Primer | Sequence (5’-3’) |
| --- | --- | --- |
| miR-29b-1 | P1 | F: AAGTAAGAGAGYGAA  R: TAAAAACCRCCCCCA |
| miR-29b-2 | P1 | F: TGGTTAGGTTTTTTG  R: AACTTATCCCRTACC |
|  | P2 | F: GTAGGAGTTGYGGTA  R: CACCTATCTCTACTT |

F: forward; R: reverse
